# Validation strategies for target prediction methods

**DOI:** 10.1093/bib/bbz026

**Published:** 2019-04-09

**Authors:** Neann Mathai, Ya Chen, Johannes Kirchmair

**Affiliations:** 1 Department of Chemistry, University of Bergen, Bergen, Norway; 2 Computational Biology Unit (CBU), University of Bergen, Bergen, Norway; 3 Center for Bioinformatics (ZBH), Department of Computer Science, Faculty of Mathematics, Informatics and Natural Sciences, Universität Hamburg, Hamburg, Germany

**Keywords:** target prediction, polypharmacology, model validation, data bias, classification, performance metrics

## Abstract

Computational methods for target prediction, based on molecular similarity and network-based approaches, machine learning, docking and others, have evolved as valuable and powerful tools to aid the challenging task of mode of action identification for bioactive small molecules such as drugs and drug-like compounds. Critical to discerning the scope and limitations of a target prediction method is understanding how its performance was evaluated and reported. Ideally, large-scale prospective experiments are conducted to validate the performance of a model; however, this expensive and time-consuming endeavor is often not feasible. Therefore, to estimate the predictive power of a method, statistical validation based on retrospective knowledge is commonly used. There are multiple statistical validation techniques that vary in rigor. In this review we discuss the validation strategies employed, highlighting the usefulness and constraints of the validation schemes and metrics that are employed to measure and describe performance. We address the limitations of measuring only generalized performance, given that the underlying bioactivity and structural data are biased towards certain small-molecule scaffolds and target families, and suggest additional aspects of performance to consider in order to produce more detailed and realistic estimates of predictive power. Finally, we describe the validation strategies that were employed by some of the most thoroughly validated and accessible target prediction methods.

## Introduction

Fueled by the growing amount of chemical and biological data, the availability of powerful phenotypic screening technologies [1], and a shift in small-molecule drug discovery from the ‘one drug one target’ paradigm to ‘polypharmacology’ [2–5], *in silico* methods for the prediction of the biomacromolecular targets of small molecules have become one of the most intensely researched areas of cheminformatics in recent years. These methods are useful not only for the discovery of new medicines but also in the repositioning of existing approved drugs [6–9].

Target prediction methods are typically pair-input problems, in that they classify a query compound and a biomacromolecule pair as an interacting (positive) or a non-interacting (negative) pair. One categorization of target prediction methods, based on the types of data used, classifies methods into three overarching approaches: ligand-based, structure-based and chemogenomic approaches [10, 11]. Ligand-based approaches make predictions based on the similarity principle, which states that similar ligands (in the context of this review, small molecules) are likely to have similar targets. These methods typically make use of a variety of molecular descriptors to quantify and compare the physicochemical properties of small molecules. They do not rely on structural information on biomacromolecules. Their applicability domain is limited primarily by the available chemical and biological data. Structure-based approaches, such as ligand docking, use structural data on biomacromolecules as the main source of information to make predictions. They are generally more computationally expensive than ligand-based methods, and their primary limitations are defined by the availability of relevant target structures and accuracy of scoring functions. Chemogenomics approaches (or proteochemometric approaches) are defined here as methods that combine information from both ligands and targets to make their predictions [10–12].

There are several publications discussing techniques that can be used in validating target prediction models [13–20]. However, among the many recently published reviews on *in silico* target prediction, only few include a discussion of validation strategies [6, 10, 11, 21–26]. With this review we aim to provide a comprehensive reference of strategies for the validation of target prediction models. The review begins with a discussion of data partitioning schemes that are used to train and test models to measure their performance, highlighting their appropriateness and limitations. This is followed by an analysis of the metrics that are used to measure this performance and of established benchmark data sets. Building up on these components, we point out strategies to obtain more realistic estimates of the performance of target prediction models that account for the biases present in the underlying reference data. Finally, we describe the validation strategies that were employed by some of the most thoroughly validated and accessible target prediction methods.

## Strategies for validating target prediction methods

Validation primarily serves two purposes: the selection of an optimal model and the evaluation of its generalized predictive performance [13, 14]. Model selection is commonly a result of an iterative model building process, during which models based on various algorithms and parameters are built on a training set and validated on a testing set. This validation procedure is generally referred to as internal validation. While often used as the sole means to report on the performance of models, internal validation is insufficient to determine the predictive performance as the iterative modeling procedure may introduce a bias toward the properties of the testing data and hence result in an overestimation of model performance. Data that are blinded to the model development process should therefore be used, in a process known as external validation, to obtain a more realistic representation of generalized performance [13]. As part of an external validation process, the training set may be further divided into a construction set (data used to train and parameterize the model) and a validation set (data used for the internal validation to optimize the model), while the testing set is held back for performance assessment [13]. With data in place to train and test the model, the metrics used to measure the performance during the testing need to be considered next. The choice of how a method was validated (that is the data partitioning schemes used for the validation) and how its performance was measured (the metrics used) are therefore essential in understanding the reported performance.

### Data-partitioning schemes

**Figure 1:**
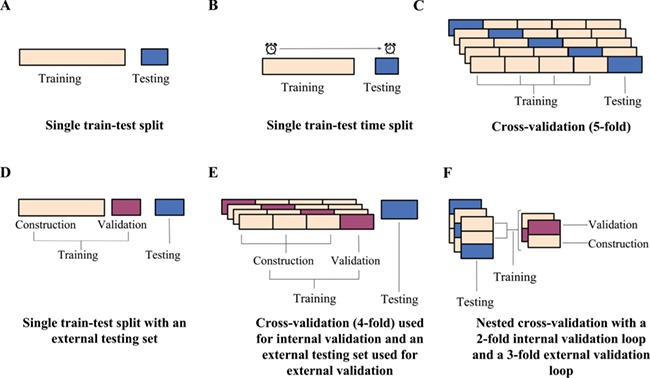
Illustrations of example data partitioning schemes: (**A**) a single train–test split, (**B**) a single train–test split of chronological data, (**C**) a 5-fold CV scheme, (**D**) a single train–test split into construction and validation sets for internal validation and an external testing set for external validation, (**E**) a 4-fold CV scheme used for internal validation with a testing set reserved for external validation and (**F**) a nested CV scheme with a 2-fold loop for internal validation and a 3-fold loop for external validation.

In the simplest case, models can be trained on one set of data and tested on another set created by random selection (Figure 1A). Such a single train–test split procedure is only effective if the training and testing sets are sufficiently large, diverse and representative of the parameter space [13, 14, 20]. However, as the limited amount of available data usually does not allow for large testing sets, the resulting test statistics may, to some extent, be an artifact of how the data were split and not an indicator of generalized performance [13, 14, 16, 18, 25]. Instead of random selection, a single split of the data into a training and a testing sets may alternatively be prepared using a time-split approach, where the model is trained on data compiled before a given date and tested on data generated later (Figure 1B). The time-split approach simulates a real-world scenario where a finalized model is put to use and new interactions are predicted [17]. Martin *et al.* [27] proposed a ‘realistic split’ approach, where compounds are clustered based on chemical similarity to mirror the exploration of new chemical scaffolds over time. In the realistic split approach, the larger compound clusters form the training set (~75% of the total number of compounds), while the remaining smaller clusters and singletons (~25%) are reserved for the testing set. The authors showed that when predicting activities of high throughput screens, a single 75:25 train–test split reported over-optimistic performance results when the split was created using a random sampling (as the compounds in the testing set were similar to the training set). In contrast, their sampling approach provided more realistic performance estimates.

**Figure 2:**
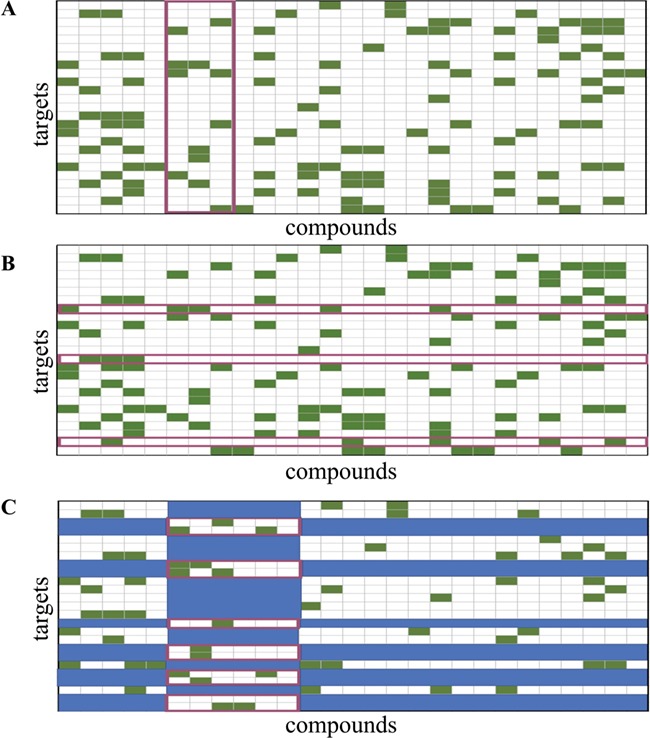
Examples of CV-testing folds designed to have (**A**) all data points involving specific queries within 1-fold (points inside the purple box), (**B**) all data points involving specific targets within 1-fold (points inside the purple box) and (**C**) all data points involving the components of query compounds–target pairs within one testing fold (points inside the purple boxes). The data points covered by the blue boxes are omitted from both training and testing data during the CV round involving the purple boxed data as the testing set, and the remaining data points are used as the training set. Interacting pairs are shown in green while (putative) non-interacting pairs are shown in white (adapted from Pahikkala *et al.* [25]).

To get a more robust estimate of how a model generalizes, cross-validation (CV) schemes have emerged, which partition the data in multiple ways to increase the variation in the training and testing data and to reduce the influence of how the data is split on the resulting testing statistics. A simple CV procedure is the *n*-fold CV, which involves randomly partitioning the data into *n* partitions and iteratively selecting each partition as the testing data while training the model on the remaining partitions (Figure 1C). The result is *n* models and *n* testing statistics, the latter of which are then averaged to give a more realistic estimate of a model’s performance [15, 19]. When *n* is equal to the number of observations, the scheme is known as the leave-one-out CV (LOOCV), with each observation playing the role of the testing set once. LOOCV is known to produce over-optimistic estimates of performance in the current context as there is a high likelihood of finding similarity between the testing molecule and the training set [13]. Therefore, typically a 5- or 10-fold CV scheme is chosen where the observations are divided into 5 or 10 folds, respectively. The folds for an *n*-fold CV are often created through random sampling. Pair-input prediction methods however are known to perform better when the tested pairs contain small-molecule or target components that are present in the training data, as such randomly generated folds for validation may produce over-optimistic performance results [16, 18, 25]. Alternative sampling methods, like stratified sampling, aim to address this issue by constructing folds with desired representations. For stratified sampling, data are first divided into the different output strata (positive or negative interactions for example) and are then randomly selected from the strata so that the desired ratio of observations is represented in the folds [14]. The folds for a CV performance assessment may also be designed to ensure that all interaction pairs involving a particular compound, compound cluster (i.e. structurally related compounds) (Figure 2A), a target (Figure 2B) or even molecule–target pairs (Figure 2C) are assigned to the same fold. These types of schemes are useful to estimate the accuracy of a method with compounds or targets with limited prior knowledge [25]. As schemes with such designed folds are likely to have fewer or no similar components between the training and testing data, the performance will be lower than that measured with a standard *n*-fold CV [16, 18, 25]. In order to give a more thorough estimation of predictive performance, it is therefore recommended that the results obtained from standard *n*-fold CV are compared to those obtained from more challenging designed-fold testing scenarios [11, 18, 25].

Most computational approaches require parametrization (e.g. the value of *k* in a k-nearest neighbour model) via iterative optimization, during which different values of the parameters are explored so as to minimize the prediction error. The repeated use of the identical training and testing sets from a single train–test split for this optimization procedure is likely to result in selection bias. That is, the optimized models may be biased towards the properties of the specific testing data [13, 14]. In cases where CV is used not only to estimate the performance of a model but also to determine the best parameters for the final model, the CV is first repeated over the different values of the parameters so as to minimize the CV error, and the parameters with the lowest validation error rates are selected for the final optimal model [14, 15]. Due to the limitations of data utilized for the development of target prediction models (such as implicit biases, data imbalance and incomplete interaction knowledge), the performance of a model determined through internal *n*-fold CV is often over-optimistic because of selection bias [18, 25]. Therefore, the performance results of this internal validation should not be considered as a rigorous estimate of the performance of the selected model. Instead, external validation should be used to evaluate the performance of the method once the model has been selected [14]. However, using a single testing set reserved for external validation (Figure 1D and E) may still produce performance statistics that are not reflective of the generalized performance but are an artifact of the testing and training split and requires the testing set to be withheld from the model [13].

Nested CV has consequently emerged as a scheme to perform external CV and better estimate unbiased performance (Figure 1F) [13–15]. In nested CV, two CV loops are run: an inner ‘internal validation’ CV loop is used for model selection and parameter optimization, and an outer ‘external validation’ loop is used for model evaluation. In the inner loop, models are trained using construction data and tested using validation data over all unique parameter values. The parameters that produced the lowest internal CV error are then used to build models for the external CV loop, where models are trained on the training set and tested on the testing set. As the testing set has remained independent of the parameter selection process, the external CV errors, often presented as an average error, are a more realistic estimate of the generalized error of the model [13–15]. It is important to note that with each iteration of the outer loop, the combination of parameters may be different due to the nature of the data in the internal loop that was used to optimize them. Nested CV does however provide the best estimate of performance [11, 14].

Often, as is the case with all the validation schemes described, even when using the data in the testing set for external validation, a final model, with parameters unchanged, is trained on the full data. The performance measures therefore do not evaluate this final model but the process of building the model. These measurements are dependent on how the data are split into the training and testing sets [13–15]. Repeated CV and repeated nested CV, to allow for data variance by resampling the folds over each repetition, have thus been recommended as a means of converging on true performance [14]. Repeated validation, commonly known as bootstrapping, is resampling the training and testing sets and repeatedly calculating performance metrics many times over. This iterative process allows for the calculation of the variation and confidence intervals of the performance metrics. Krstajic *et al.* [14] propose a repeated nested CV scheme, where the internal and external validation loops each have 50 repetitions, and the lowest and highest error metric, in addition to the average error metric, are reported to show the variance in the method’s performance. They recommend using random *n*-fold CV for the internal loop and stratified CV for the external loop when using repeated nested CV to develop and evaluate a model [14].

In addition to reporting statistical metrics generated from the above validation schemes, illustrative case studies are also often reported to highlight the performance of a method. However, reporting on just a few case studies is not a sufficiently rigorous approach to determine a model’s performance [26]. Ideally, large-scale experimental studies would need to be conducted that allow not only thorough validation but also a demonstration of a method’s potential impact. However, due to cost, such large-scale studies are generally not carried out.

### Performance metrics

**Figure 3:**
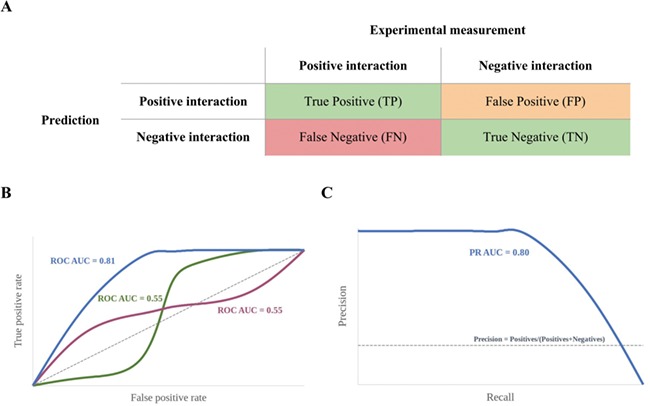
(**A**) A binary classification confusion matrix with the four categories of prediction (FPs may include putative false positives); (**B**) ROC curves: the closer the curves are to the top left-hand corner, the better. AUC values alone may be deceptive as a lack of correct early predictions may be offset by an increased number of correct predictions later, leading to high AUC values. This scenario is shown by the green and purple curves. (**C**) Precision-recall curve: the closer the curve is to the top right corner, the better the model’s performance.

In its most basic form, target prediction can be regarded as a binary classification problem: a small molecule either interacts with a biomacromolecule (a positive interaction) or it does not (a negative interaction). Based on this premise, a common evaluation technique is to complete the confusion matrix. The confusion matrix shows how the predictions made by a method on a testing data set (in the current context, data on small molecules) compare to the known recorded interactions of these compounds. A two-class confusion matrix consists of a set of four tallies of the prediction results: the number of true-positive (TP), true-negative (TN), false-positive (FP) and false-negative (FN) predictions (Figure 3A). Metrics to describe the performance of a method are then calculated using these entries. Importantly, the FP predictions may in fact include undiscovered or unreported interactions and may therefore be more precisely referred to as assumed FP predictions. Performance metrics generally do not account for this kind of missing data, and it is therefore more appropriate to consider this component as potential FP predictions.

Two simple measures calculated from the confusion matrix are the model’s sensitivity (SE) and specificity (SP). SE (also recall or TP rate) quantifies the model’s ability to detect positive interactions and is the fraction of how many of the known positive interactions are identified by the target prediction method

(1) \begin{align*} SE= \frac{TP}{TP+ FN} \end{align*}

SP, or TN rate, quantifies the model’s ability to detect negative interactions and is the fraction of how many known, or assumed, negative interactions are identified by the prediction method

(2) \begin{equation*} SP= \frac{TN}{TN+ FP} \end{equation*}

Precision (PR), or positive predictive value, quantifies how many of the predicted interactions are known interactions for a compound or a set of compounds

(3) \begin{equation*} PR= \frac{TP}{TP+ FP} \end{equation*}

Accuracy (ACC) is a basic metric of the overall performance of binary classifiers that quantifies the proportion of correct predictions

(4) \begin{equation*} ACC=\frac{TP+ TN}{TP+ TN+ FP+ FN} \end{equation*}

A limitation of this metric is that it does not account for data set imbalance, which is a ubiquitous issue in target prediction, where data are often made up of a small number of recorded ligand–target interactions (positive class) and a large number of observed or assumed non-interactions (negative class). In this context, a target prediction method that correctly predicts most non-interactions but fails to identify known positive interactions would obtain high ACC values, despite its inability to correctly identify the targets of small molecules [28].

A metric that does consider the proportion of all classes in the confusion matrix and therefore addresses the issue of imbalanced data is the Matthews Correlation Coefficient (MCC). The MCC quantifies the correlation between the predictions and their true value

(5) \begin{equation*} MCC=\frac{\left( TP\ \cdot\ \mathrm{T}N\right)-\left( FP\ \cdot\ FN\right)}{\sqrt{\left( TP+ FP\right)\cdot \left( TP+ FN\right)\cdot \left( TN+ FP\right)\cdot \left( TN+ FN\right)}}\end{equation*}

MCC values range from −1 to +1, with +1 indicating perfect prediction, 0 a prediction as good as random and −1 a prediction that is in total disagreement with the measured data. Although the MCC is regarded as one of the most robust measures of the quality of binary classification, it is rarely used in target prediction. In the special case when a model predicts very few FPs and very few TPs at the same time, the MCC value will be deceptively high [29].

Other correlation metrics, such as Cohen’s kappa (κ) are sometimes used to measure the performance of a classifier. Cohen’s kappa measures the similarity between two sets of classifications (in this case, the predicted classes and the known classes for interactions). Kappa quantifies how much better or worse a classifier is compared to random chance [30–32].

All metrics discussed so far aim at quantifying the ability of classifiers to discriminate interacting from non-interacting pairs of small molecules and biomacromolecules. However, rather than only predicting categories, most target prediction models return a score or probability that is used to rank predicted (non-) interactions. The ability of a target prediction method to recognize interacting pairs of ligands and targets and to rank them early in the hit list (‘early recognition’) is a key parameter for the goodness and value of such models. A straightforward and often used measure of early recognition is the top-*k* metric, which quantifies the percentage of compounds for which a defined number of known interactions is ranked among the top-*k* positions. Statements such as ‘for X% of all tested molecules, at least one known target was ranked among the top *k* targets’ are used to report performance. Note that the top-*k* metric obviously depends on an arbitrary cut-off (the *k* value) and the number of targets considered for ranking, and it does not account for the statistical likelihood of random pick [33].

The receiver operating characteristic (ROC) curve is used to determine early enrichment, without an earliness cut-off. The ROC curve is an easily interpretable plot of the TP rate (SE) on the y-axis versus the FP rate (1-SP) on the x-axis, and it is drawn by calculating the cumulative positives and negatives as one moves down a rank-ordered list (Figure 3B) [34]. The closer a ROC curve approaches the top left corner of the graph, the better the rank-ordered list is, since TPs are identified early on, achieving early enrichment. A ROC curve that approaches the diagonal represents the random classification of small molecule and target pairs. Parts of the ROC curve located below the diagonal indicate a performance that is worse than random ranking.

The ROC curve considers both the correctly classified positive values (SE on the y-axis has TP in the numerator) and the correctly classified negative values (1-SP has TN in the denominator) and is therefore a good measure for balanced data sets [28, 35]. In contrast, the precision-recall curve plots PR (which has TP in the numerator) on the y-axis versus recall (which also has TP in the denominator) on the x-axis and is therefore ideal at visualizing how well positives appear at the top of the ranking, particularly when the data set has an imbalanced distribution between positives and negatives (Figure 3C) [28]. Unlike the ROC curve, the closer the precision-recall curve is to the top right edge, the better. The random classification of small molecule and target pairs results in a precision-recall curve that approaches the straight line, where PR is equal to the fraction of positives in the data set. Parts of the curve located below this line indicate a performance that is worse than random ranking.

The goodness of a classifier, as reflected by ROC and precision-recall curves (and others), can, in part, be quantified by the area under the curve (AUC). AUC values are bound between 1, for ideal models, and 0, for models that make predictions that are entirely the opposite of the recorded results. To draw conclusions about a model’s early recognition ability, both AUC values and the original curve need to be considered, as models that perform differently with respect to early enrichment may have the same AUC since a lack of early recognitions may be offset by later recognitions (Figure 3B) [36, 37].

As the AUC metrics are not sensitive to early recognition, the robust initial enhancement (RIE) was developed as a single parameterized metric based on the enrichment factor (which is the factor by which known interactions are ranked more often within the top-*k* predictions compared to random selection of *k* predictions)

(6) \begin{equation*} RIE\left(\alpha \right)=\frac{\sum_{i=1}^n{e}^{-\alpha {r}_i/N}}{{\left\langle {\sum}_{i=1}^n{e}^{-\alpha {r}_i/N}\right\rangle}_{random}} \end{equation*}

The RIE uses a decreasing exponential weight to calculate how much better a ranked list of interactions is compared with the list with random distribution of the positive and negative targets [38, 39]. The RIE value is dependent on the early cut-off exponential parameter (α) and the ratio of positive interactions in the list, the product of which is the exponent component of the metric. RIE values therefore cannot be compared, unless the same cut-off and proportion of actives are present, making it harder to compare different methods [34, 39].

The Boltzmann-enhanced discrimination of ROC (BEDROC) metric, developed by Truchon *et al.* [34] for easier comparison, is the RIE metric scaled between 0 and 1, with 1 implying perfect prediction

(7) \begin{equation*} BEDROC\left(\alpha \right)=\frac{RIE\left(\alpha \right)-{RIE}_{min}\left(\alpha \right)}{RIE_{max}\left(\alpha \right)-{RIE}_{min}\left(\alpha \right)} \end{equation*}

A BEDROC value of 0.5 is when the observed cumulative distribution function (the cumulative number of actives versus the number of predictions in a rank-ordered list) has the same shape as the cumulative distribution function exponentially parameterized by the ⍺ parameter. This allows BEDROC scores with the same α parameter to be compared. The BEDROC metric is therefore more useful in discriminating a method’s early recognition capabilities than an AUC due to the exponential weights and allows for easier comparison than the RIE metric [34, 39].

### Benchmark data sets for target prediction

Benchmark data sets can be useful for the comparative assessment of target prediction approaches. However, due to the complexities involved in compiling high-quality representative data sets, only few have been reported to date. One of the more widely used [22, 40, 41] benchmark data sets for target prediction is the Yamanashi data set [42], which was compiled from different sources and comprises 5127 drug–target interactions of 932 drugs and 989 targets for G protein-coupled receptors (GPCRs), ion channels, enzymes and nuclear receptors. Koutsoukas *et al.* [43] published a benchmark data set consisting of ~100 k compounds compiled from the ChEMBL database [44] used to compare the performance of different machine-learning algorithms [43]. Peón *et al.* [45] compiled two benchmarking data sets for their comparative study of ligand-centric methods for target prediction, one with 183 k active compounds with activities (EC50, Ki, Kd or IC50) below 10 μm and one with 147 k active compounds with activities below 1 μm. The data set used for externally testing SwissTargetPrediction has been made available for use as a benchmark [46]. Most recently, Wang and Kurgan [47] compiled and curated a data set from several different databases, consisting of 449 compounds, 1469 targets and 34 k interactions. One of a very few sources offering a complete data matrix of compounds tested against an array of different proteins is the kinase data set published by Davis *et al.* [48], which comprises 72 diverse kinase inhibitors measured against 442 kinases and was suggested by Pahikkala *et al.* [25] as a high-quality data set for testing target prediction methods. Two benchmark data sets specifically designed for testing structure-based methods have also been reported [49].

## Strategies for obtaining more realistic estimates of model performance

Rigorous validation schemes, involving external validation, in combination with information-rich performance metrics, quantify how well a method has generalized. However, the data employed for target prediction models are usually heavily biased. In opposition to reality, for example, chemical databases commonly have an overrepresentation of known actives compared to known inactives [10, 24, 26]. Established drug targets are much better represented by the available chemical, structural and biological data than other biomacromolecules [11, 50]. Additionally, the synthesizability of compounds and the fact that medicinal chemistry tends to generate congeneric series of compounds lead to significant biases in the represented scaffolds [11, 51]. These biases are a natural result of the drug-development environment and lead to concentrations of information on certain targets and scaffolds.

Some targets are more challenging to predict than others due to the specific properties of individual targets or the structural and functional relationships between the biomacromolecules covered by a target prediction model. For example, due to its large and malleable ligand-binding site and no clear pharmacophoric requirements, cytochrome P450 (CYP) 3A4 binds to a broad variety of ligands [52, 53]. These properties mean that, despite the availability of a substantial body of structural, chemical and biological data, CYP3A4 is a particularly challenging target to address for both ligand and structure-based methods [54]. It is also much more difficult for target prediction methods to discriminate small-molecule activity among structurally and/or functionally related biomacromolecules. That is, it will be more challenging to correctly predict a protein kinase inhibitor’s selectivity profile for kinases than it is to understand whether the compound will also bind to a certain GPCR. For all these reasons, the number of biologically tested compounds or the number of crystal structures by which a target is represented in the reference data is not the only factor that determines how difficult it is for a model to make predictions for a specific molecule or target.

Given these data biases and challenges, it is clear that averaged performance metrics have limited significance as they obfuscate the predictive power of a method across queries and target classes. In fact, the individual characteristics of the targets and molecules covered by a target prediction model and by the testing set will determine the measured performance of a model. It is therefore generally not possible to directly compare results on model performance obtained from different studies as these usually use different data for model training and testing.

**Figure 4:**
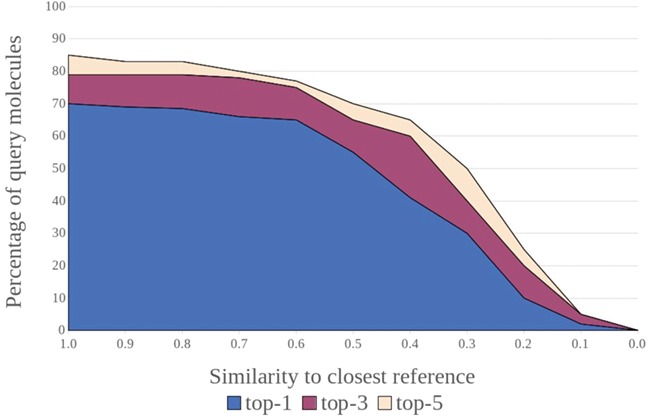
Success rates for a target prediction model (e.g. percentage of compounds for which at least one known target was ranked among the top 1, top 3 and top 5 positions) versus the maximum similarity between the individual query compounds and their closest related compounds in the reference data. Such plots are powerful tools to visualize a method’s capacity for inter- and extrapolation and help with the definition of the applicability domain.

To obtain a more realistic representation of the performance of a target prediction model, a number of measures may be carried out to ameliorate the impact of the data and model biases:

(i) A combination of metrics and methods that are more robust against the imbalance [10, 11, 55, 56] between known actives and inactives in the data set (e.g. precision-recall curve, PR AUC and the MCC) should be used for model testing. It is also useful to present the confusion matrices of the performance tests, so that further metrics may be calculated and used to compare methods.
(ii) For any averaged performance metrics, their minima, maxima and distributions of values should be reported. A repeated validation scheme to calculate ROC curves would be useful in evaluating performance, as an average ROC curve with its confidence interval can be shown for assessment.
(iii) Stratified sampling may be applied to construct more realistic data sets that mimic the real world, for training and testing. Caution must be exercised to ensure that oversampling of a class does not result in a model that is overfit.
(iv) External data should be used for the evaluation of model performance.
(v) In addition to a standard CV or nested CV, the performance of a model should also be evaluated using the various designed folds to establish performance estimates under conditions where there is no knowledge of the query molecule or target (Figure 2) in the training data.
(vi) From a ligand perspective, building on established concepts in applicability domain research [45, 57–61], a weighted performance metric should be derived that is an improvement on the averaged metrics that quantify generalized performance. Such a metric would account for the difficulty of predicting the targets of individual query molecules as a function of the structural similarity between the query and the training instances (in the case of structure-based approaches, the similarity to the closest bound ligand may be used). Graphical approaches can be powerful tools to visualize such relationships, as shown by the example in Figure 4. These strategies can provide a better understanding of a method’s capacity for inter- and extrapolation and help with the definition of the applicability domain.
(vii) Performance metrics could also take into account the complexity of the (known) bioactive chemical space for the individual targets (in particular, in terms of size and diversity) as it is indicative of the number of ligand-binding pockets and subpockets, their size, shape, flexibility and specificity (in terms of pharmacophoric requirements).
(viii) From a target perspective, a weighted performance metric could be used that takes into account the coverage and complexity of the conformational phase space relevant to ligand binding. Parameterizing such a performance metric is a non-trivial task, as in most cases the relevant conformational phase space remains unknown to a large extent. As an approximation, tools such as SIENA [62] may be used to automatically align protein-binding sites and quantify structural deviations among them.
(ix) The druggability of a target, which is the likelihood of being able to modulate a target’s activity with a small molecule [63, 64], may also be an indicator of how difficult it is, in particular for a docking algorithm, to make predictions for a specific target. Buried ligand-binding sites featuring hydrogen bond donors and acceptors are, for example, typically less challenging to address with small molecules than shallow hydrophobic interfaces on the protein surface (as often observed for protein–protein interaction interfaces) [65]. Docking algorithms show similar trends; ligand-binding sites that lack directed interactions or are solvent exposed are more challenging, for example.
(x) The structural and functional relationships between the individual targets covered by a model should also be taken into account. TP predictions of targets that are related and therefore more challenging to discriminate should be assigned a higher weight than correct predictions for targets that are distinct. Likewise, a putative FP prediction of a target that is in agreement with activity recorded for a related target should be assigned a lower weight. Putative FP predictions are cases where compounds are predicted as active on a particular target, but no bioactivity data are available to confirm or refute this prediction. Given the low likelihood of a compound being active on a random biomacromolecule, for the purpose of evaluation, the general assumption made is that the compound is indeed inactive on that target. However, in the case of closely related targets there is a good chance that a compound confirmed to be active on one target is also active on the other. Ideally, the structural similarity of targets would be assessed based on the comparison of 3D structures of the ligand-binding sites. Given the complexities involved in such comparisons, this is generally not feasible on a large scale. Instead, the sequence similarity of the protein domains involved in ligand binding may be used as a rough indication of the structural similarity of targets as perceived from a ligand’s perspective.
(xi) While there is no universal gold standard data set, evaluating a model’s performance on benchmarking data sets will allow for easier comparison among methods.
(xii) In addition to the many strategies involving statistical means, a critical discussion of representative examples can be very useful to better understand the scope and limitations of target prediction models. This could include comparing the performance of a model for well-represented versus underrepresented targets or highlighting the ability of a model to discriminate targets of a group of related biomacromolecules versus a group of distinct targets.

## Examples of how popular target prediction methods have been validated

Today, a large number of target prediction models are accessible via (mostly free) web services [2, 21, 50, 66–69]. The rigor applied in the evaluation of these methods varies greatly. For some models, their predictive power has been demonstrated by a small number of case studies (e.g. ChemMapper [70], Mantra [71, 72] and TarFisDock [73]). A substantial proportion of models have been evaluated on larger sets of data (e.g. ChemProt [74], CSNAP [75], DR. PRODIS [41], HitPick [76], Semantic Link Association Prediction (SLAP) [77], SuperPred [78] and TargetHunter [79]). Others have undergone systematic statistical validation by CV (e.g. SPiDER [80] and SwissTargetPrediction [81]). In one case, namely Similarity Ensemble Approach (SEA) [82], large-scale experimental evaluations have been reported. We describe four examples of popular target prediction models that have undergone some of the most thorough validation experiments reported so far.

**SEA** (http://sea.bkslab.org) is an early ligand-based method that predicts the targets of small molecules based on their similarity to ligand sets of a reference database [82]. SEA has been tested through multiple rounds of prospective validation [82, 83]. The largest study reported so far is by Novartis and included the analysis of 1241 predicted interactions for 656 approved drugs. Of the predicted interactions, 348 were retrospectively verified. Further 694 predictions were experimentally tested, of which 48% were confirmed and 46% were disproved [84]. A number of studies have since used SEA [85–87] to identify, for example, the targets of the small molecule ogerin as the adenosine A_2A_ receptor and of SLV 320, an adenosine A_1_ antagonist, as an inhibitor of GPCR68 [88]. SEA has undoubtedly had the largest impact and use of all target prediction methods, and this can be attributed to its early development and the large-scale experimental testing by Novartis that is not typically feasible.

**SwissTargetPrediction** (http://www.swisstargetprediction.ch) is a ligand-based similarity method that uses both 2D fingerprints and 3D shape, combined in a logistic regression, to predict the likely targets of small molecules [81]. SwissTargetPrediction covers more than 2600 targets from five organisms (human, mouse, rat, cow and horse) and is arguably one of the most thoroughly statistically validated target prediction methods in existence [46]. The method also suggests the orthologs and paralogs of the predicted biomacromolecules as potential targets. SwissTargetPrediction was evaluated by a standard and two designed 10-fold CV runs. For the 1st designed CV run, molecules with similar scaffolds were incorporated into the same CV fold to estimate the performance of the method when the method is used with structurally distinct ligands [81]. This experiment was repeated using an additional 2nd filter to group molecules that were tested in the same assay within the same fold, thus reducing the probability of a comparison of ligands from the same series [81]. For all the CV experiments, the folds were created to have 10 times as many negative interactions as positive ones, with the number of negative interactions supplemented by randomly pairing ligands and targets with no known positive interactions together. As expected, the performance of the method was lower for the designed CV runs (distinct scaffolds ROC AUC 0.979; distinct scaffolds and assays ROC AUC 0.932) than it was for the standard CV (ROC AUC 0.994). The effects of ligand properties (e.g. number of heavy atoms and lipophilicity) on the prediction accuracy were also investigated. In order to estimate the performance on new molecules, a 2nd external testing set that was composed of 213 molecules with 346 positive and 278 new interactions recorded in the consecutive version of the ChEMBL database. The testing set was expanded with randomly assigned ligands and targets to ensure that there were five times as many negative interactions than positive interactions in the testing set. On these data, the model obtained a ROC AUC of 0.87.

**SPiDER** (http://modlabcadd.ethz.ch/software/spider/) [80] is a ligand-based method that utilizes self-organizing maps in combination with ‘fuzzy’ CATS pharmacophore descriptors [89] and Molecular Operating Environment (MOE) descriptors [90]. Validation of the method was carried out through a stratified 10-fold CV during which a prediction was considered successful if all known targets of a query were predicted within a defined significance threshold. The results from the CV were combined to calculate the ROC curve and ROC AUC value of 0.92 [80]. The capacity of SPiDER to predict the biomolecular targets of small molecules was demonstrated by a number of studies involving synthetic molecules [80, 91–94] as well as natural products [92, 95].

**SLAP** (http://cheminfov.informatics.indiana.edu:8080/slap/) is a network-based method that uses data from 17 sources and a semantic network linking the diverse and related data types (chemical compound, substructure, side effect, chemical ontology, target, disease, gene family, tissue, pathway and gene ontology) [77]. A chemical compound and a target are considered to be associated based on the defined path patterns, which include characteristics such as the length and the type of nodes involved in the paths between them. To evaluate the model’s performance, four testing sets were compiled with known drug–target pairs from DrugBank and random drug–target pairs (serving as negative interactions), such that the ratio of positive and negative interactions was 1:1, 1:4, 1:8 and 1:12. The ROC AUCs (about 0.92 for all sets) and the precision-recall curves were reported for these tests, along with the performance measures by target class. SLAP was also evaluated on 23 confirmed drug–target pairs that were identified with SEA, and it was found that the method is not capable of identifying cross-boundary targets. In addition, SLAP was evaluated on 444 drug–target pairs recorded in MATADOR [96] (and not represented in the network) and successfully identified 170 of these interactions with high confidence.

## Conclusions

A plethora of *in silico* models have become available in recent years and are increasingly utilized to guide efforts to identify the biomacromolecular targets of small molecules. While the modeling approaches have come of age, there is room for further improvement in the validation of the methods. Ideally, target prediction methods would be tested in large-scale, prospective studies, but high expenses in terms of costs and time are, in general, prohibitive to such efforts. Therefore, developers and users rely on robust retrospective (statistical) analyses. One of the most elaborate efforts of retrospective validation was published for SwissTargetPrediction, where a standard CV, two CVs with designed folds and a time-split approach were executed and analyzed in combination.

One of the most obvious deficits of current approaches to retrospective validation is their limitation to the global assessment of model performance, which can vary substantially for individual query molecules and targets as they are represented in the reference data to different extents. Here, the development of weighted scoring functions that account for the challenges involved in predicting the interaction of specific pairs of small molecules and biomacromolecules is desirable and urgently needed. A 2nd major limitation of current retrospective studies is their lack of comparability, which is a result of a lack of established, high quality, benchmark data sets and the complexities involved in the validation of target prediction models. It will take time for both of these issues to be resolved, but there are several immediate steps that can be taken to obtain more realistic estimates of model performance. As a minimum requirement, any target prediction method should undergo a systematic statistical validation. In particular, it is important for parameterized models to undergo external validation, and the results obtained from this test should be discussed with respect to the results obtained from internal validation. The discussion of representative test cases is desirable, e.g. the ability of a model to discriminate bioactivities of small molecules on structurally distinct targets in contrast to structurally related targets.

We submit that current reports on the performance of models often miss to convey the implications of the outcomes of statistical tests on the usefulness of target prediction methods under real-life conditions. In contrast to the common assumption made during model validation, investigators will most likely have prior knowledge of some biological properties of a compound. Armed with their expert knowledge they will often be able to identify false predictions. For the same reason, FP predictions on targets structurally related to the real target of a small molecule (e.g. predictions of activity on CYP1A2, whereas the compound actually is an inhibitor of CYP3A4 and not CYP1A2) can be useful as they may point researchers into the right direction, even though current validation approaches would commonly consider these predictions as false. It is also likely that investigators will have knowledge of several structurally related compounds exhibiting the same kind of biological activity rather than a singleton. By using multiple structurally related compounds as queries the signal-to-noise ratio can be improved. On the downside, in a real-life scenario, compounds of interest are likely to be more distant to the training data than the average compound of the testing set, which makes observing the applicability domain of a model an important issue.

Overall, we believe, and the recent reports in the literature show, that *in silico* models have become powerful tools to aid the identification of the mode of action of small molecules. We should not expect target prediction methods to generally be able to correctly rank the targets of a compound of interest among the top 1 or top 3 out of several hundreds or thousands of biomacromolecules. However, we are on a good track of developing models that are able to provide valuable guidance to experimentalists in their efforts to confirm the relevant targets of small molecules and to point out if a compound of interest is outside of the applicability domain of a model. This is a qualitative improvement to the challenging task of mode of action identification, and the increasing availability of chemical and biological data will lead to a further boost of theoretical methods for target prediction.

## Notes

Key Points

- *In silico* models have become important and powerful tools to efforts to identify the biomacromolecular targets of small molecules.
- Commonly followed strategies in assessing the performance of target prediction approaches do not adequately account for the heavy biases present in the chemical and biological data utilized for training and testing.
- A number of immediate measures can be taken to obtain more realistic estimates of the performance of target prediction models.
- New metrics that weigh the difficulty of individual predictions are urgently needed, as are benchmark data sets enabling the comparative performance analysis of target prediction methods.

## Notes

**Neann Mathai** is a PhD student at the Department of Chemistry and the Computational Biology Unit (CBU) of the University of Bergen (UiB) and also affiliated with the University of Hamburg (UHH). Her research focuses on the development and application of computational methods for target prediction.

## Notes

**Ya Chen** is a PhD student at the Center for Bioinformatics (ZBH) of the UHH. Her research focuses on the development and application of computational methods for the prediction of the biomacromolecular targets of natural products.

## Notes

**Johannes Kirchmair** is an associate professor in bioinformatics at the Department of Chemistry and the CBU of the UiB. He also is a group leader at the ZBH. His research activities focus on the development and application of *in silico* methods for the prediction of the biological activities, metabolic fate and toxicity of xenobiotics.
